# Gastrointestinal issues predominate among side effects of medium-chain triglyceride consumption in humans

**DOI:** 10.3389/fnut.2026.1829954

**Published:** 2026-08-19

**Authors:** Marc Scheller, Marius Frenser, Juliet Valembois, Tobias Fischer

**Affiliations:** Center for Nutrition and Therapy (NuT), University of Applied Sciences Muenster, Münster, Germany

**Keywords:** abdominal pain, adverse events, diarrhea, gastrointestinal, medium-chain fatty acids (MCFAs), medium-chain triglycerides (MCTs), side effects, tolerability

## Abstract

**Introduction:**

Medium-chain triglycerides (MCTs) are widely used in clinical and nutritional contexts due to their rapid metabolism and therapeutic potential. However, concerns regarding their tolerability persist, particularly with respect to gastrointestinal side effects. This systematic review examines the occurrence and characteristics of side effects following oral MCT consumption.

**Methods:**

In accordance with the PRISMA guidelines and the Cochrane Handbook, a systematic literature search was conducted in PubMed and Web of Science. Studies reporting side effects following oral, gastric or enteral MCT administration were included. Data on study design, population characteristics, MCT formulation, dosage, and reported side effects were extracted and synthesized. Side effects were analyzed quantitatively or qualitatively in studies using non-standard reporting formats. Risk of bias was assessed using the RoB 2 tool for randomized studies and the ROBINS-I tool for non-randomized studies.

**Results:**

Thirty three studies comprising 1,100 participants were included. Gastrointestinal symptoms were the most frequently reported side effects, particularly diarrhea, abdominal pain, discomfort, and nausea. While these symptoms were often described as mild, moderate to severe reactions and dropouts due to side effects were not uncommon, especially at higher doses. Substantial heterogeneity in study design, dosing strategies, and reporting practices limited comparability. Temporal aspects of side effects and long-term tolerability were insufficiently investigated, and evidence regarding the influence of MCT composition and co-administration strategies on tolerability remained inconclusive. A high proportion of studies reported industry involvement, and risk-of-bias assessment indicated that 26.3% (RoB 2)-−78.6% (ROBINS-I) of the included studies were at high/serious risk of bias.

**Conclusions:**

Gastrointestinal side effects represent a consistent and clinically relevant limitation of MCT use. Standardized methodologies and targeted research are needed to better understand and mitigate these effects, thereby improving the safety and applicability of MCT-based interventions.

## 1 Introduction

Medium-chain triglycerides (MCTs) are receiving increasing attention in various fields of medical and dietetic research, including the treatment and prevention of dietary conditions (1). MCTs are defined as triglycerides based on medium-chain fatty acids (MCFAs) with chain lengths of 6–12 carbon atoms (2, 3). Unlike long-chain triglycerides, MCTs are absorbed largely independently of cholecystokinin-stimulated bile secretion and do not require incorporation into chylomicrons for transport. After hydrolysis, MCFAs are transported directly via the portal vein to the liver, where they are rapidly oxidized for adenosine triphosphate (ATP) production or converted into ketone bodies (2, 4–6). MCTs are much more ketogenic than long-chain triglycerides (7, 8), with caprylic acid (C8:0) exhibiting the strongest ketogenic potential, followed by capric acid (C10:0), while lauric acid shows a comparatively weaker ketogenic effect (9). The medical and clinical applications of MCTs are diverse and include, among other things, the management of gastrointestinal disorders associated with fat malabsorption (10), their use in weight management strategies (11), and therapeutic approaches for neurological conditions such as epilepsy and Alzheimer's disease (12). In addition, MCTs play an important role in the dietary treatment of rare inborn errors of metabolism, particularly disorders of long-chain fatty acid oxidation, where they provide a rapidly oxidizable energy source (13). Another emerging area of application for MCTs is the enhancement of athletic performance, particularly through improvements in endurance and energy availability as their rapid oxidation and ketogenic properties may support metabolic efficiency and energy metabolism during prolonged or high-intensity exercise (14, 15). Their popularity has also breached the mainstream consumer market, MCTs now being sold over the counter and in online shops as a dietary supplement that may aid with weight loss or exercise (16). Despite the broad range of potential applications and increasing public interest, the current body of evidence on MCT efficacy, safety, and tolerability of MCTs is limited and partly conflicting, warranting further systematic research (17–20). Many commercial claims seem to exceed the strength of the current scientific evidence, highlighting the need for cautious interpretation. As early as 1971, Huttenlocher et al. (12) reported gastrointestinal complaints following the ingestion of 39 ml of MCTs, which could be mitigated by emulsifying and slow consumption. During further examinations evaluating the consistency of MCTs' beneficial effects in humans (21–23), side effects like gastrointestinal disturbances have regularly been observed and reported (24–27). Reported side effects are linked to rapid absorption, osmotic activity, accelerated intestinal transit and reduced cholecystokinin stimulation (28–30). In a systematic review of seven studies involving MCTs, side effects were documented in only two studies (28.6%), although these effects were clinically meaningful (19). Existing scoping reviews on MCTs primarily focus on metabolic and functional aspects, including ketogenic effects in clinical applications, while safety considerations are addressed only briefly and without systematic evaluation (16, 31). In these reviews, side effects are typically reported unsystematically and without differentiation regarding frequency, severity, or influencing factors such as dosage, fatty acid composition, or population characteristics. Consequently, a comprehensive and structured assessment of MCT-related side effects and their determinants is currently lacking. Therefore, the present systematic review provides a structured up-to-date evaluation of MCT-related side effects, with a primary focus on occurrence, characteristics, and determinants, thereby addressing a clearly defined gap in the current literature.

## 2 Materials and methods

The systematic literature review was conducted in accordance with the methodological standards of the PRISMA guideline (Preferred Reporting Items for Systematic Reviews and Meta-Analyses) (32), taking into account the methodological recommendations of the Cochrane Handbook (33). The review protocol was not prospectively registered.

### 2.1 Search strategy, selection criteria and screening

A systematic literature search was performed in the electronic databases PubMed and Web of Science. The database searches included records published up to March 31, 2026. A sensitive search strategy was created with the help of selected keywords and terms as well as the use of appropriate operators. The search strategy combined the concept of medium-chain triglycerides with side effects using the Boolean operator “AND.” Within each concept, relevant search terms and synonyms (e.g., “discomfort,” “nausea,” and “diarrhea” for side effects) were combined using the Boolean operator “OR.” In addition, corresponding keywords and Medical Subject Headings (MeSH; PubMed) were included to comprehensively capture relevant literature (search queries see Supplementary Table 1). Inclusion and exclusion criteria are presented in Table 1 and were defined using an adapted PICOs scheme (population, intervention(s), comparator(s), and outcomes) tailored to the objectives of this systematic review (34). Studies were included if they investigated human populations (P), regardless of age, gender, ethnicity, health status, or geographical location, receiving dietary MCTs, MCFAs, or MCFA-containing products administered orally or via gastric or enteral routes, including MCT-based ketogenic diet protocols (I). No specific comparator (C) was required. Outcomes (O) comprised reported side effects plausibly associated with MCT intake. Eligible study designs (S) included clinical studies and trials (e.g., randomized controlled, comparative, and multicentre studies) published in English. Animal and *in vitro* studies, studies that did not assess or provide any information on side effects, and studies involving parenteral or intravenous MCT administration were excluded. In accordance with the recommendations of the Cochrane Handbook (35), the results were imported into the literature management program Citavi (version 7.0; Lumivero, Denver, CO, USA) and duplicates were removed. Titles and abstracts of the remaining studies were screened against the predefined eligibility criteria, after which full texts of potentially relevant studies were retrieved and assessed. In addition, reference lists of included studies were screened to identify further relevant literature. Study selection, data extraction (2.2), and risk-of-bias and conflict of interest assessment (2.3) were performed by one reviewer and subsequently verified by a second researcher from the same institution.

**Table 1:** Inclusion and exclusion criteria for the systematic literature review.

Unit	Inclusion criteria	Exclusion criteria
Article type (PubMed)	Clinical study, clinical trial, comparative study, multicentre study, randomized controlled study article	Any other type of article not listed as an inclusion criterion
Document type (Web of Science)	Article	Any other type of document not listed as an inclusion criterion
Research areas (Web of Science)	Gastroenterology and hepatology, nutrition and dietetics, endocrinology and metabolism, pharmacology and pharmacy, toxicology, medicine, general and internal, neurosciences and neurology, public, environmental and occupational health, sport sciences, physiology, biochemistry and molecular biology, food science and technology, agriculture	Any other type of research area not listed as an inclusion criterion
Language	English	Any other language not listed as an inclusion criterion
Population	All human studies (regardless of age, gender, ethnicity, pre-existing health conditions, geographical location)	Animal studies and *in vitro* studies
Intervention	Dietary intervention with or administration of MCTs or MCFAs or MCFA-containing products such as oils, formulas or supplements consumed in oral form or administered gastric or enteral, as well as MCTs supplemented as part of a ketogenic diet protocol	Intervention without MCTs or MCFAs, parenteral administration of MCTs, intravenous administration of MCTs, use of Triheptanoin as sole MCTs source
Outcome	Side effects and adverse events assessed and reported, including studies explicitly reporting the absence of side effects or adverse events	Studies not assessing or reporting side effects or adverse events

### 2.2 Data extraction and data analysis

The following data were extracted in Microsoft Excel (Microsoft Office Professional Plus 2016, version 1808; Microsoft Corporation, Redmond, WA, USA) according to the recommendations of the Cochrane Handbook (36): study characteristics (author, title, year, DOI, conflict of interest/funding), population (number, sex, age, dropout, preexisting health conditions), intervention (chain length of MCTs (C8/C10), intervention time, dosage, form of administration), and outcome (diarrhea, abdominal pain/discomfort, nausea, vomiting, constipation, fatigue, dizziness, bloating and flatulence, headache, unspecified gastrointestinal issues). Studies reporting side effects as absolute numbers or percentages were included in the quantitative synthesis. For these studies, the proportion of participants experiencing a side effect was calculated using the number of participants who received at least one MCT intervention. Where reported, intervention-specific sample sizes were used. Studies assessing side effects exclusively through questionnaire-based assessments of symptom severity, burden or inconvenience without reporting frequencies were analyzed separately in a qualitative approach. In addition to overall dropout rates, dropouts explicitly attributed to side effects were extracted and analyzed separately. Descriptive statistics, including minimum and maximum values, mean values, medians, and standard deviations were calculated to characterize data distribution.

Findings across the included studies were structured and grouped according to predefined characteristics, such as the form of MCT administration, co-administered components and formulation, dosage, intervention duration, and the type and frequency of reported side effects. No weighting by study size was applied due to data heterogeneity. Study-level estimates were calculated individually and subsequently summarized across the studies. When multiple intervention arms were present, reported side effects were summarized. Due to heterogeneity, a systematic data analysis of literature was performed.

### 2.3 Risk-of-bias and conflict of interests assessment

Revised Cochrane Risk-of-Bias Tool for Randomized Trials (RoB 2) was applied for quality assessment (33). The Cochrane Excel implementation for individually randomized parallel-group trials (ROB2_IRPG_betav9) was used to guide the evaluation (37). According to the RoB 2 guidance, overall risk of bias was classified as “low” when all domains were rated as low risk. A judgment of “some concerns” was assigned if at least one domain was rated as “some concerns” and none as high risk. Overall risk was considered “high” if at least one domain was rated as high risk or if multiple domains were rated as “some concerns,” indicating substantial methodological limitations (38). Non-randomized studies were assessed using the Risk Of Bias In Non-randomized Studies—of Interventions (ROBINS-I) tool. Overall risk of bias was categorized according to the ROBINS-I guidance as low, moderate, serious or critical risk of bias. The overall judgement corresponded to the highest risk level identified across the individual bias domains (39).

In addition, a predefined classification of conflicts of interest was applied, based on the extent of industry involvement and its potential influence on study design, conduct, and reporting. Accordingly, the extracted conflicts of interest were classified into three predefined categories: (1) low risk (no reported conflicts), (2) moderate risk (industry funding, company employment of at least one researcher, or industry cooperation) and (3) high risk (industry involvement in study design or publication).

## 3 Results

### 3.1 Study selection

**Figure 1:**
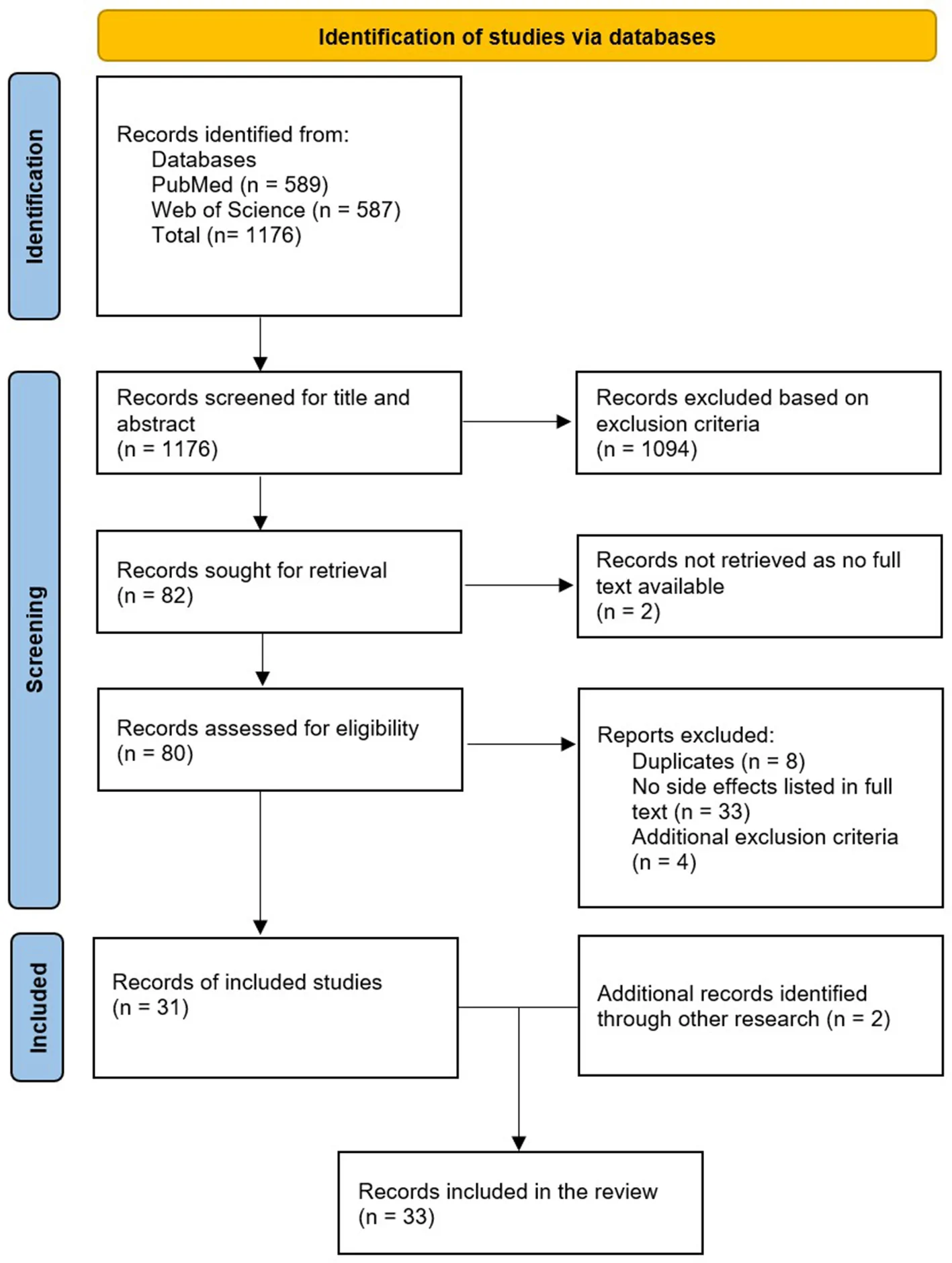
Modified flow chart of the literature search and selection based on the PRISMA flow diagram for systematic reviews (32).

A total of 1,176 results were identified (PubMed: *n* = 589; Web of Science: *n* = 587). Following title and abstract screening, 1,094 studies were excluded due to an inappropriate thematic focus (e.g., intervention or research area). During full text screening, studies were excluded if they did not report side effects (*n* = 33), were duplicates (*n* = 8), or employed MCT administration methods that were not aligned with the predefined inclusion criteria (*n* = 4). The remaining records (*n* = 31), together with additional studies identified through reference screening (*n* = 2), resulted in a total of 33 studies included in this review (Figure 1).

### 3.2 Characteristics of the studies

The studies were published between 1984 and 2026, with 22 studies (66.7%) being published in the last 10 years (7, 26, 27, 40–57), and 13 studies (39.4%) in the last 5 years (7, 26, 40, 42, 48, 51–58). Thirteen (39.4%) of the studies were conducted in or affiliated with researchers from Europe (7, 26, 28, 40, 45, 49, 50, 52, 59–63), 11 studies (33.3%) in America (27, 46, 48, 51, 54, 55, 58, 64–66), five studies (15.2%) in Asia (42, 44, 47, 53, 56), three studies (9.1%) in Oceania (41, 43, 67) and one study (3.0%) in Africa (68). The included studies encompassed a range of study designs, including uncontrolled interventions, randomized controlled designs, as well as prospective, retrospective, and crossover approaches, comprising a total of 1,100 participants. Sample sizes were smaller in studies assessing acute or performance-related outcomes and larger in longitudinal dietary interventions, with a mean of 33 ± 35 participants (range: 6–152) and a median of 19. On average, 45 ± 29% of participants were female. Five studies (15.2%) included exclusively male participants (42, 56, 60, 67, 68), whereas three studies (9.1%) recruited only female participants (40, 48, 65). The mean age across all studies was 33.6 ± 21.9 years, ranging from a minimum mean age of 4.0 years (54) to a maximum mean age of 68.7 years (46). Twelve studies (36.4%) reported age ranges spanning at least 10 years within their study populations (44, 46, 47, 49–51, 53, 57–59, 62, 64), while four studies (12.1%) did not report mean or individual age data (60, 62, 65, 66). Fourteen studies (42.4%) included healthy participants, while the remaining studies investigated clinical populations. The most frequently studied clinical conditions were epilepsy, followed by Alzheimer's disease, essential tremor, obesity, Parkinson's disease, and post-concussion syndrome. Detailed population characteristics are provided in Supplementary Table 2.

### 3.3 Interventions and controls

**Figure 2:**
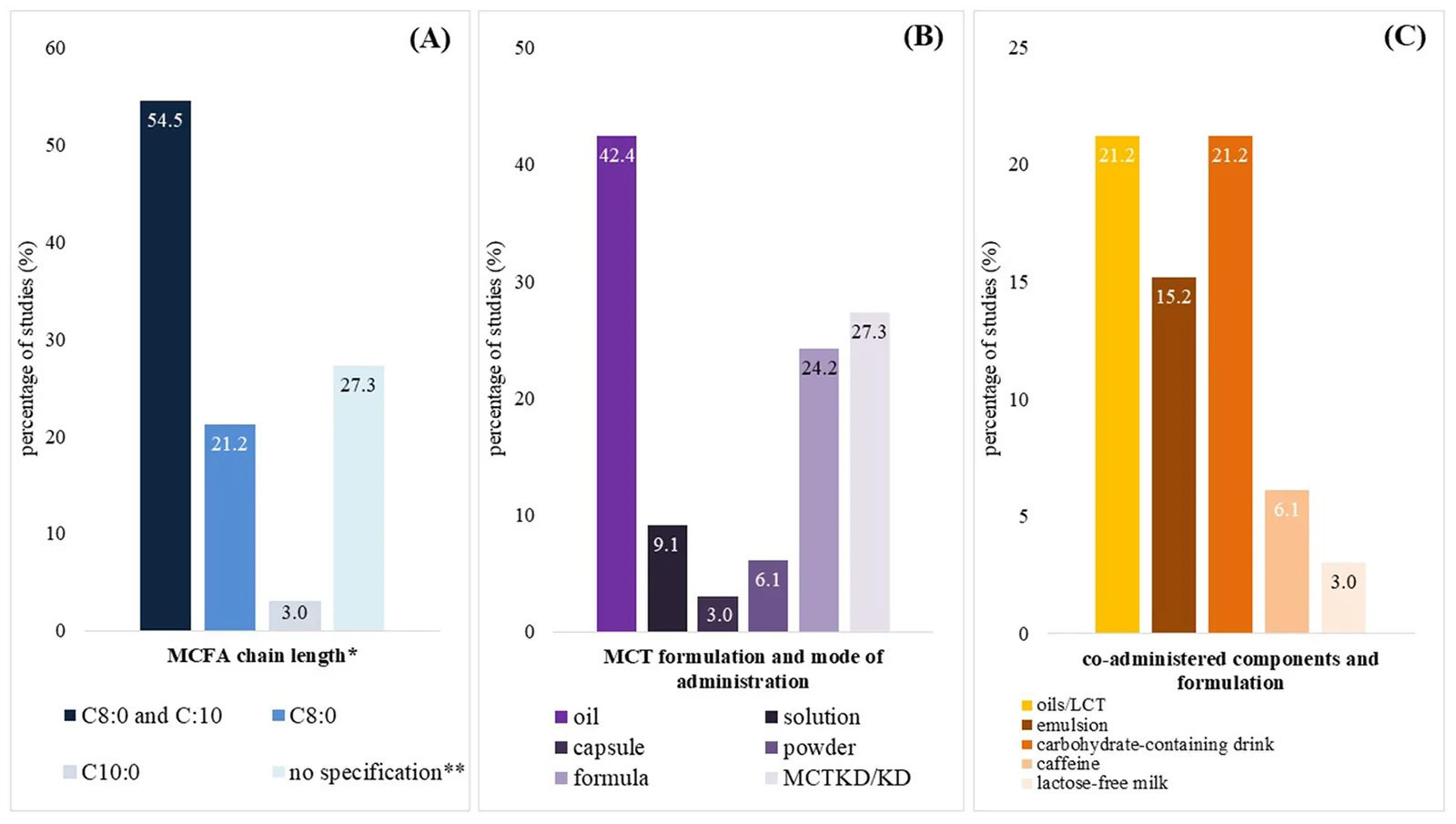
Distribution of MCT interventions characteristics across included studies, including **(A)** MCFA chain length, **(B)** formulation and mode of administration, and **(C)** co-administered components and formulation. Values are expressed as percentage of studies. MCTKD, MCT ketogenic diet; KD, ketogenic diet. *One study investigated both C8:0 and C10:0, administered either individually or in combination, and is therefore represented in three of the categories outlined above (27). **Due to MCT ketogenic diets or pharmaceutical or medical product.

Across the included studies, MCT dosing varied substantially with respect to both timing and amount. Most interventions followed a longitudinal design (57.6%, *n* = 19) (41–45, 47–54, 57, 58, 61, 62, 65, 66), with intervention duration ranging from 5 days (42) to 24 months (50) (median duration: 20 days). In these studies, MCT intake typically ranged from 35% (41) to 60% (61) of daily energy intake or from 26% (49) to 80% (65) of total fat-derived calories. One study could not be included in this comparison due to insufficient reporting of caloric intake and MCT dosage (57). Single-administration protocols accounted for 33.3% of studies (*n* = 11) (7, 26, 27, 40, 46, 55, 56, 59, 60, 63, 64) and involved doses ranging from 4 mg/kg (62) to 0.5 g/kg (26) bodyweight or absolute amounts between 8 g (46) and 85 g (60); this category also included studies with repetitive single administrations. A smaller proportion of studies applied continuous administration over defined time periods, such as minutes or hours (9.1%, *n* = 3) (28, 46, 67). In one study (3.0%, *n* = 1), MCTs were administered intraduodenally via transnasal catheter (28), while all remaining studies used oral administration. The most common form of MCT administration was MCT oil, followed by MCT-centered dietary regimens, predominantly MCT ketogenic diets with varying fat ratios, and formula-based interventions. Co-administered components and formulation strategies varied widely and included carbohydrate-containing drinks, alternative carriers, and emulsification approaches, which have been suggested to influence tolerability (50, 51, 60, 63). The characteristics of MCT interventions across the included studies are summarized in Figure 2.

### 3.4 Risk of bias assessments

**Figure 3:**
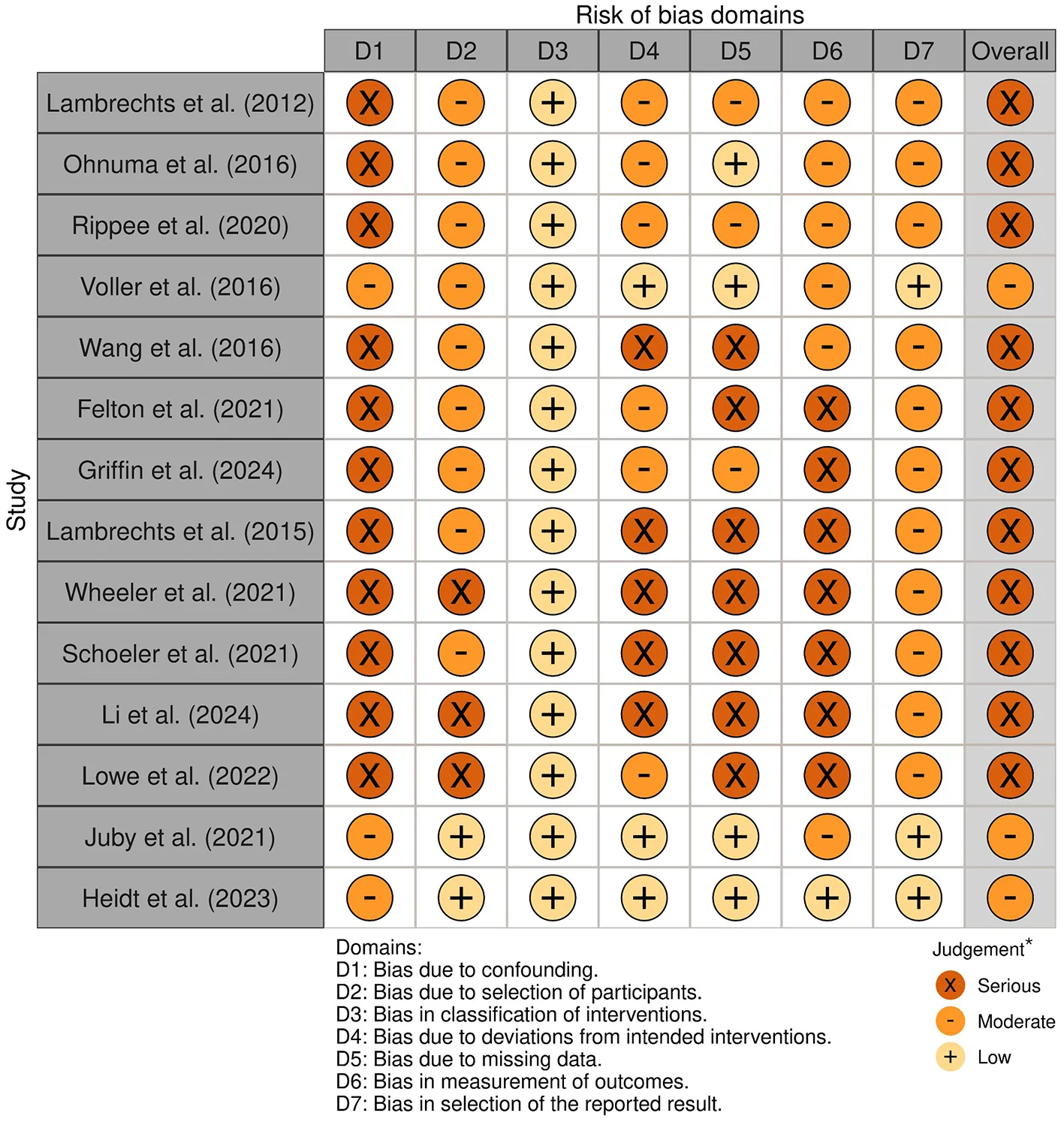
Risk of bias assessment across non-randomized studies using the ROBINS-I tool (39). *No study was assessed as having a critical risk of bias.

**Figure 4:**
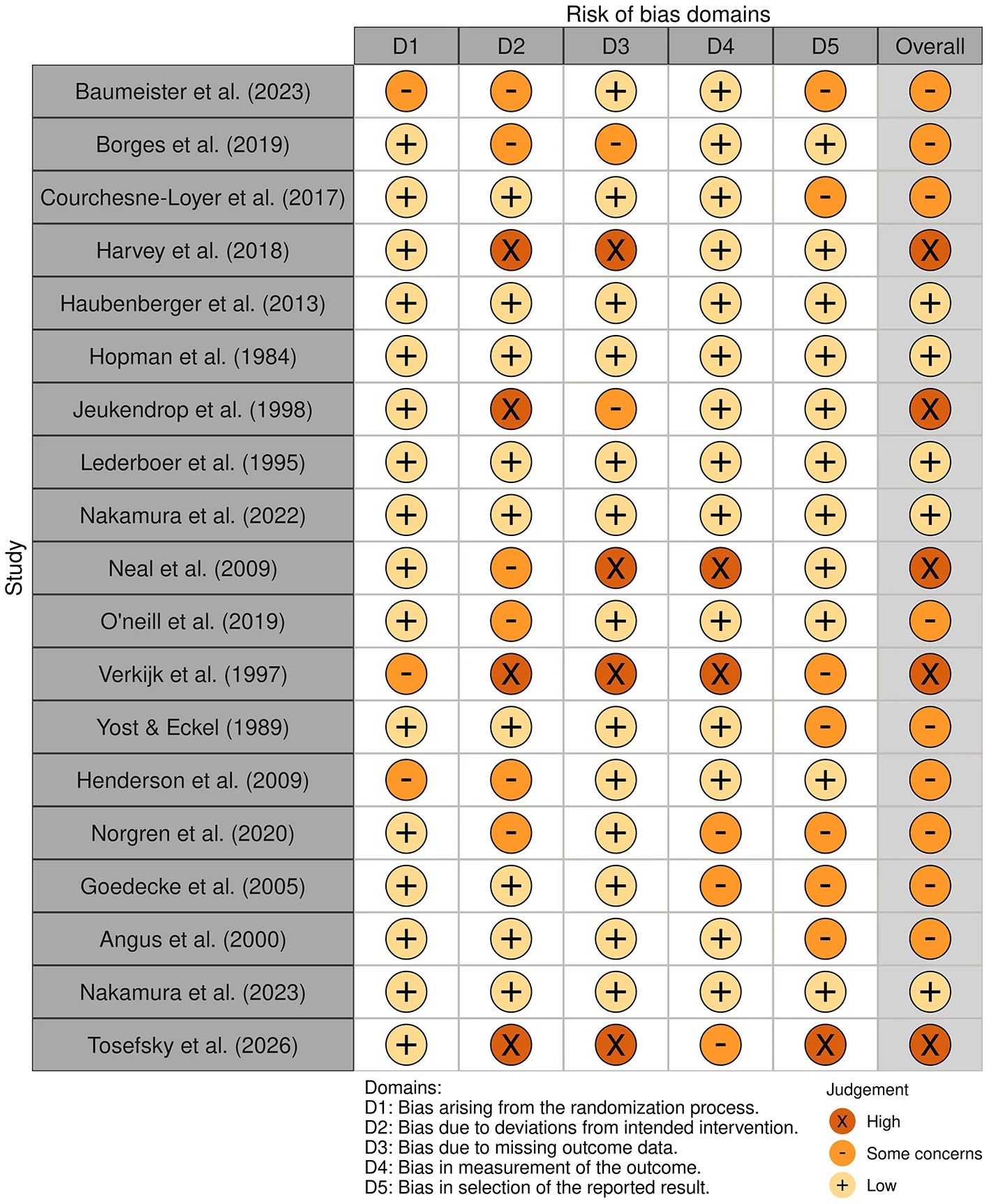
Modified visualization of the risk of bias assessment across randomized studies using the Rob 2 tool (37).

A serious risk of bias was identified in 11 of the 14 non-randomized studies (78.6%), whereas the remaining studies (21.4%) were rated to have a moderate risk of bias (see Figure 3). No study was rated to have a critical risk of bias. The classification was primarily driven by the absence of appropriate comparator groups, insufficient control for confounding, high attrition rates, deviations from intended interventions, open-label designs combined with subjective outcome assessments, and the lack of prespecified analysis plans or preregistered protocols, thereby limiting causal inference. The remaining 19 studies were randomized controlled trials (RCTs). Of these, five (26.3%) were rated as low risk of bias, nine (47.4%) as having some concerns, and five (26.3%) as high risk (see Figure 4). Within the RCTs, concerns most commonly related to insufficient reporting of allocation concealment and, in crossover designs, inadequate description of washout procedures. Studies employing objective outcome measures and appropriate statistical analyses were generally classified as low risk. Overall, 16 of the 33 included studies (48.5%) were classified as having a serious/high risk of bias.

### 3.5 Conflicts of interest

Regarding conflicts of interest, 12 studies (36.4%) did not report any apparent conflicts and were therefore classified as low risk (27, 28, 40, 47, 50, 53, 55, 57, 58, 60, 62, 63). Seventeen studies (51.5%) reported industry affiliation and were classified as having a moderate risk of bias (7, 26, 41–45, 48, 49, 51, 54, 59, 61, 64, 65, 67, 68). Four studies (12.1%) were categorized as high risk, as the affiliated or sponsoring company was directly involved in study design, data interpretation, or publication decisions (46, 53, 56, 66). Across the included studies, six (18.2%) involved one or more investigators who were current or former employees of the affiliated or supporting company (42, 45, 49, 52, 56, 66). Several other studies were conducted in collaboration with companies primarily operating in the pharmaceutical sector. These studies received industry funding, and one or more investigators had previously received grants, honoraria, or compensation for advisory or consultancy services from the respective companies (7, 26, 41, 43, 44, 48, 51, 54, 59, 61, 64, 65, 67).

### 3.6 Outcomes (side effects of MCTs intake)

Four studies (12.1%) assessed side effects using questionnaire-based assessments of symptom severity or inconvenience, including Likert-type or validated gastrointestinal scales (7, 43, 56, 60). One study reported mean symptom severity scores across all participants, which were highest during the early phase of MCT administration and decreased over the course of the intervention (43). Two further studies employed graded scales of symptom severity or inconvenience and reported low to moderate gastrointestinal complaints (7, 60). Another study used the validated Gastrointestinal Symptom Rating Scale and found no substantial changes between baseline and post-intervention measurements (56).

**Figure 5:**
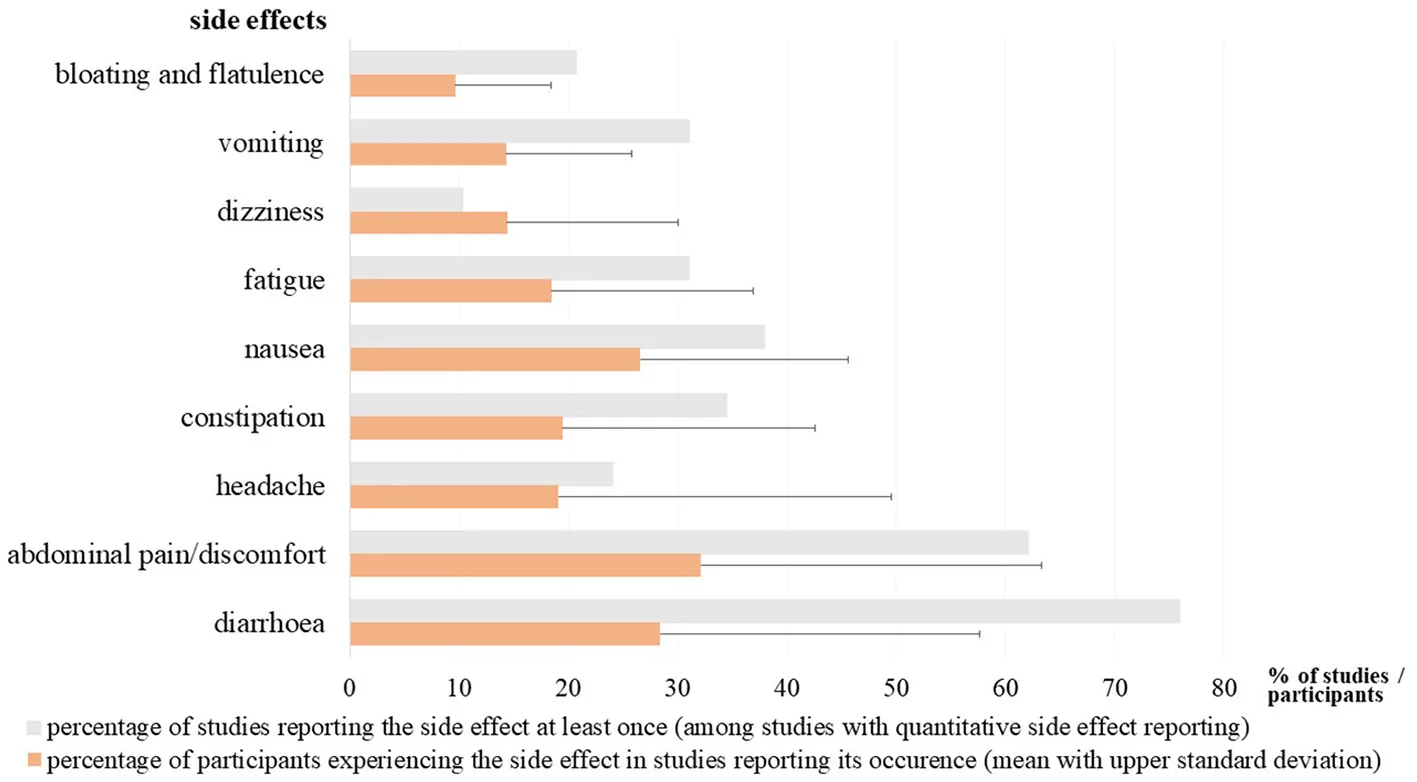
Frequency of reported side effects across included studies compared with experienced side effects among participants.

The remaining 29 studies (87.9%) reported side effects quantitatively, using absolute numbers or percentages, and were included in the subsequent analyses. Diarrhea was the most frequently reported side effect (22 studies, 75.9%) (26–28, 40, 41, 44, 45, 47–50, 52–55, 57–59, 61, 63, 66, 67), followed by abdominal pain or discomfort (18 studies, 62.1%) (26, 27, 40–42, 44–46, 48–50, 52–55, 61, 63, 65), nausea (11 studies, 37.9%) (26, 27, 40, 41, 45, 48, 49, 52, 54, 55, 58) and constipation (10 studies, 34.5%) (41, 47, 48, 50–54, 61, 64). Less frequently reported symptoms included vomiting (26, 47, 50–54, 61, 67), fatigue (40, 46, 50, 53, 55, 57, 58, 62, 64) (each nine studies, 31.0%), headache (seven studies, 24.1%) (27, 40, 41, 46, 55, 57, 64), bloating/flatulence (six studies, 20.7%) (41, 44, 45, 49, 52, 65), and dizziness (three studies, 10.3%) (26, 40, 46). Six studies (20.7%) reported unspecified gastrointestinal symptoms (49, 57, 62, 65, 66, 68). Overall, gastrointestinal complaints predominated across all studies. All remaining 29 studies reported at least one gastrointestinal effect. The proportion of affected participants varied widely, with diarrhea ranging from 4% (49) to 100% (60, 63) across studies. While diarrhea was most frequently reported, abdominal pain and discomfort (32.1 ± 31.1%) and unspecified gastrointestinal issues (34.8 ± 27.6%) affected a higher proportion of participants within their respective study cohorts. Bloating and flatulence were least prevalent (9.6 ± 8.8%; see Figure 5).

Nine of the 33 studies (27.3%) reported information on the temporal characteristics of side effects, including their onset and/or duration (7, 26–28, 46, 55, 60, 67, 68). Symptoms typically occurred within 2 h of intake and resolved within 30 to 360 min. Persistence beyond 1 day was reported only after the administration of a total of 90 ml MCT within a single day (28). Longitudinal studies generally lacked detailed temporal reporting. Most long-term studies relied on participants self-reporting via questionnaires or diaries, which were generally assessed at predefined time points.

**Figure 6:**
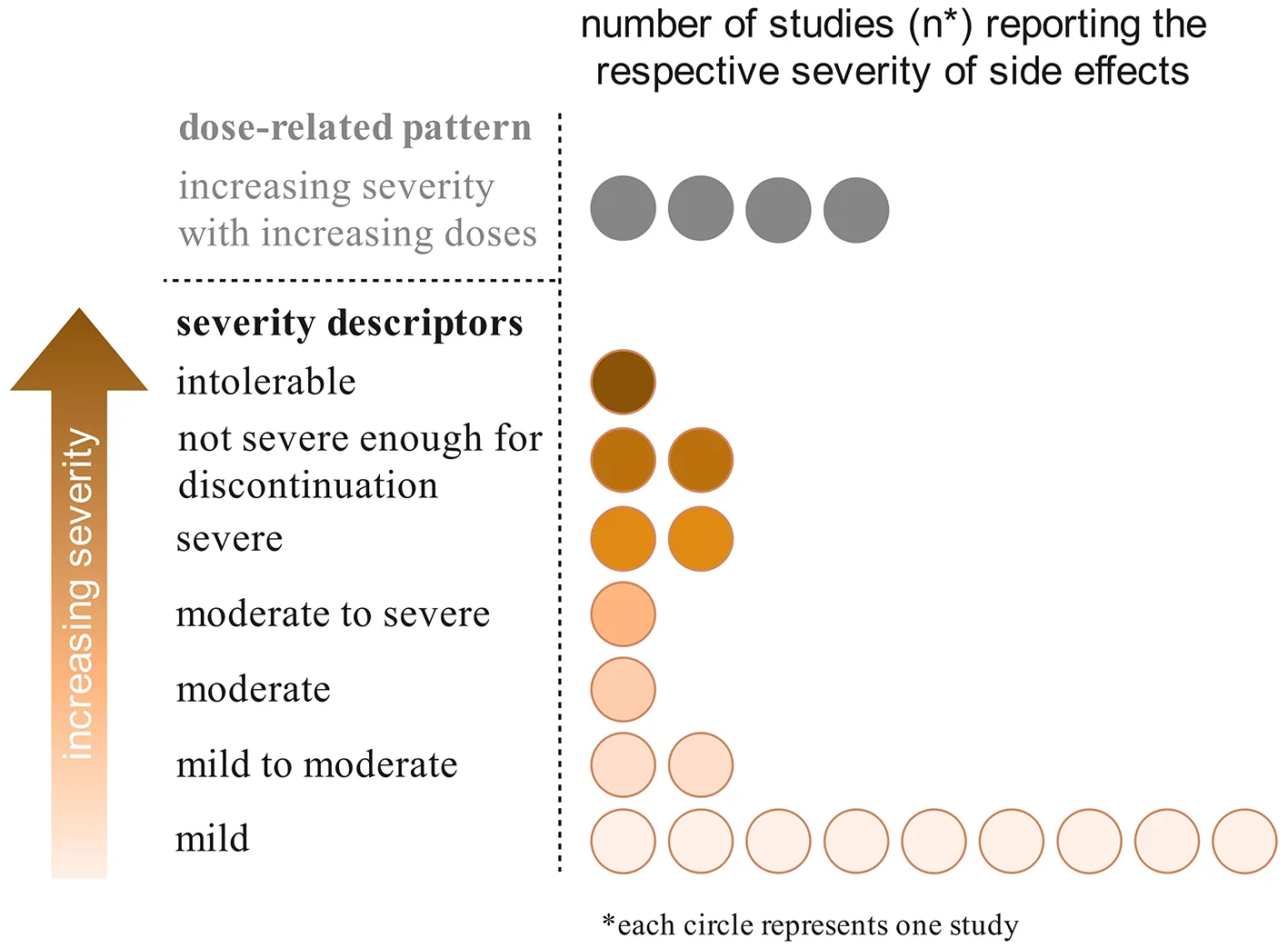
Distribution of reported side effects severity levels across the included studies.

20 of the 33 studies (60.6%) reported qualitative information on the severity of side effects, most commonly categorizing symptoms as mild, moderate, or severe (7, 27, 28, 41–43, 45, 46, 49, 51, 55–57, 59, 60, 64–68). Of these, nine studies (45.0%) described side effects as mild or predominantly mild. Except for one study (57), severity was not quantified and was instead reported using qualitative descriptors such as “mostly” or “largely” (7, 41, 42, 46, 51, 59, 64, 65). Two studies (10.0%) reported mild to moderate symptoms (49, 60), one study (5.0%) described generally moderate side effects (43), and one study (5.0%) reported moderate to severe symptoms (67). In one study (5.0%), high-dose administration of 90 ml of MCTs within a single day was discontinued for ethical reasons due to intolerable side effects (28). Two studies (10.0%) reported that side effects did not necessitate discontinuation of the intervention (26, 55). Four studies (20.0%) reported increasing side effects with increasing MCT doses (27, 28, 45, 55). Figure 6 illustrates the distribution of side effect severity across studies.

Attempts to improve tolerability yielded inconsistent results. 150 mg caffeine co-administration (40) and high-pressure homogenization of MCTs (27) were associated with improved tolerability. In contrast, co-administration of glucose showed 50% less side effects in one study (26), whereas other studies reported no benefit or increased side effects (7, 60, 67, 68). One study further stratified side effects according to MCT chain length and composition. When events from interventions with and without caffeine were combined, 13 side effects were reported following administration of C8, nine for C10, and five for a combined C8/C10 formulation (40).

### 3.7 Dropouts

Dropout rates varied across studies. Short-term or single-administration studies reported few or no dropouts, whereas longitudinal studies reported dropout rates of up to 77.0%, resulting in an overall mean dropout rate of 18.0 ± 23.7%. Reasons for study discontinuation included severity of side effects, difficulties adhering to the study protocol, lost to follow-up, family/personal issues, social issues, and perceived lack of efficacy. Fifteen studies (45.5%) reported no dropouts during the study period (7, 26–28, 46, 51, 52, 55, 56, 59, 60, 63, 65, 67, 68), while one study (3.0%) did not report dropout data (42). Of the remaining 17 studies (51.5%), six studies (18.2%) reported no side effect-related dropouts, with withdrawals attributed to other reasons (40, 48, 50, 54, 58, 64). In contrast, 11 studies (33.3%) explicitly reported withdrawals due to side effects (41, 43–45, 47, 49, 53, 57, 61, 62, 66). Across these 11 studies, the mean dropout rate attributable to side effects was 11.4 ± 6.8%.

## 4 Discussion

The present systematic review indicates that gastrointestinal side effects are the most frequently reported side effects associated with MCT administration, based on an up-to-date and structured synthesis of evidence spanning several decades across diverse populations, intervention protocols, and study designs. Diarrhea, abdominal pain or discomfort, and nausea were the most frequently reported complaints across diverse populations, intervention protocols, and study designs, consistent with previous reports (19, 23, 69). However, evidence on long-term tolerability remains sparse and inconsistently reported, which is of particular relevance given the increasing clinical use and popularity of MCTs. This highlights the need for more systematic and standardized investigations of their safety profile, as also emphasized by Shcherbakova et al. (70).

The influence of MCFA chain length and MCT composition on side effect profiles remains insufficiently studied. Although differential effects of caprylic acid (C8:0) and capric acid (C10:0) have been discussed in other contexts, particularly with regard to procognitive effects by Shcherbakova et al. (70), only one included study stratified side effects by chain length and composition. In that study, a combined C8/C10 formulation showed better tolerability than the isolated administration of either MCFA (40). The remaining studies did not examine this relationship precluding definitive conclusions (44, 46, 49, 55, 60, 64–66).

Similarly, the potential role of carbohydrate co-supplementation in improving tolerability remains unclear. Although suggested in previous reviews and clinical practice that co-ingestion of MCTs with meals may improve gastrointestinal tolerability (4, 70), the available data is limited, with few studies, partially overlapping designs, and often lacked appropriate comparator groups (7, 19, 60). Notably, gastrointestinal symptoms were also reported in a study administering MCTs alongside a Mediterranean diet, suggesting that concomitant food intake does not necessarily prevent gastrointestinal side effects (57). Therefore, no consistent benefit of carbohydrate co-administration can currently be concluded.

A formal dose–response analysis was not feasible, as dosing strategies, intervention designs, and outcome metrics were highly heterogeneous. Nevertheless, several studies reported that increasing amounts of MCTs were associated with a higher occurrence and severity of side effects, suggesting reduced tolerability at higher doses, particularly in the short term. Accordingly, recommendations suggest gradually increasing MCT intake, with daily amounts up to 50–100 g and single doses below approximately 30 g (17, 71). However, findings remain inconsistent, as similar gastrointestinal symptoms were reported at both high (85 g MCT) (60) and lower doses (26, 27, 44, 45, 47, 48, 51–54, 63, 66, 67). In this context, the high-intensity exercise protocol employed in the study from Jeukendrup et al. (60) may have contributed to symptom occurrence. A systematic review with meta-analysis likewise reported variable tolerability, even at higher intakes (22). Moreover, substantial inter-individual variability in both ketogenic response and side effects was observed (55). The present findings reinforce this, as side effects varied markedly even among participants receiving identical doses. The influence of individual factors, such as age, sex, or gastrointestinal health, could not be determined from the available data, as these variables were not part of the investigations. Taken together, the current evidence suggests that a uniform dosing strategy may be insufficient, and individualized, gradually titrated approaches are likely necessary to optimize both safety and tolerability of MCT interventions.

Short observation periods of only minutes to hours appear insufficient to capture delayed reactions, and the duration of reported side effects was often inconsistently reported or not reported at all. This highlights that not only the timing of onset but also the resolution of symptoms should be systematically captured through study protocols that provide sufficiently frequent and structured reporting intervals. Side effects were frequently assessed as secondary outcomes and relied primarily on self-reporting, increasing the risk of underreporting (72). As noted by Frenser et al., (19) reports of few or no side effects may reflect insufficient assessment rather than tolerability. Incomplete and inconsistent reporting, particularly regarding severity, duration, and temporal progression (73, 74), indicates a lack of standardization and suggests that current methods may underestimate the true incidence of MCT-related side effects.

Side effects were a relevant reason for participant dropout, with rates of up to 26% (41). In addition, several longitudinal studies reported high overall dropout rates, in some cases exceeding 60% (47, 49, 53, 62). Interpretation is impacted by inconsistent reporting of withdrawal reasons and incomplete differentiation between side effect-related and non-side effect-related dropouts. A randomized feasibility study investigating ketogenic diets in patients with glioblastoma reported poor retention, with only four of twelve participants completing the 3-month intervention, three of whom were assigned to the MCT ketogenic diets. Withdrawals were largely attributed to the perceived burden of the dietary regimen and concerns about quality of life rather than side effects alone (75). Likewise, a recent study on a modified MCT ketogenic diet, providing 20%−30% of total energy intake from MCTs in patients with drug-resistant epilepsy, reported declining retention rates over time (98.4%, 65.0% and 33.3% at 1, 3, and 6 months, respectively), while only 3.3% of patients discontinued due to side effects. Instead, most withdrawals were related to poor efficacy, refusal to follow the diet, or difficulties maintaining adherence (53). Together, these findings suggest that dropout in MCT interventions is often multifactorial and not solely driven by side effects.

A further consideration is the industry involvement, with a substantial proportion of studies classified as having moderate to high conflicts of interest. This may have influenced the reporting and interpretation of findings. Previous analyses also indicate that financial conflicts may contribute to underreporting of side effects (11, 20, 76).

A limitation of this systematic review is the use of a predefined but non-standardized classification of conflicts of interest, as no universally established framework currently exists. Nevertheless, the applied approach allowed a structured and transparent assessment of potential industry influence across the included studies. The completeness and consistency of reported funding sources and conflicts of interest vary across studies, potentially limiting the reliability of such classifications (77). Duplicate records were removed at a later stage of the screening process than typically recommended in PRISMA workflows; although this approach was transparently reported and did not affect the final study selection, it may have affected the representation of excluded records in the flow diagram.

## 5 Conclusions

Gastrointestinal side effects represent a consistent and clinically relevant limitation of MCT administration. Symptoms such as diarrhea, abdominal pain, and nausea were frequently reported across the included studies, suggesting that tolerability remains an important consideration in MCT interventions and application. Further research is needed to better understand, predict, and mitigate these side effects to enable an effective application of MCTs. However, substantial heterogeneity in study design, population characteristics, and side effect reporting, as well as a considerable proportion of industry involvement and the associated risk of bias, limit the interpretation of the current evidence. Future research should prioritize standardized methodologies and explicitly investigate determinants of MCT tolerability, including fatty acid composition, dosing strategies, inter-individual variability, and long-term effects.

## Data Availability

The original contributions presented in the study are included in the article/Supplementary material, further inquiries can be directed to the corresponding author.
