# Supplementary material for: Gastrointestinal issues predominate among side effects of medium-chain triglyceride consumption in humans
Source: Front Nutr. 2026 Aug 19;13:1829954. doi: 10.3389/fnut.2026.1829954 (PMC13533690; doi:10.3389/fnut.2026.1829954)
Supplement: Supplementary file 1 [file Table_1.docx]

Supplementary Material

# 1 Supplementary Tables

| **Data Base** | **Final Search String** |
| --- | --- |
| **Pub Med** | ("MCT"[tiab] OR "medium chain triglycerid*"[tiab] OR "me-dium chain fatty acid*"[tiab] OR "caprylic acid*"[tiab] OR "capric acid*"[tiab] OR "octanoic acid*"[tiab] OR "decanoic acid*"[tiab] OR "C8"[tiab] OR "C10"[tiab]) AND ("side ef-fect*"[tiab] OR "adverse react*"[tiab] OR Adverse Ef-fects[MeSH Subheading] OR "toxic"[tiab] OR "nausea"[tiab] OR "diarrhea*"[tiab] OR "diarrhoe*"[tiab] OR "vomit*"[tiab] OR "discomfort*"[tiab] OR "stomach pain*"[tiab] OR "gastric pain*"[tiab] OR "abdominal pain*"[tiab] OR abdominal pain[MeSH Terms] OR "cramp*"[tiab] OR "lack of ener-gy"[tiab] OR "headache*"[tiab] OR "safe*"[tiab] OR safe-ty[MeSH Terms]) |
| **Web of Science** | TS=("MCT" OR "medium chain triglycerid*" OR "medium chain fatty acid*" OR "caprylic acid*" OR "capric acid*" OR "octanoic acid*" OR "decanoic acid*" OR "C8" OR "C10") AND TS=("side effect*" OR "adverse react*" OR "toxic*" OR "nau-sea*" OR "diarrhea*" OR "diarrhoe*" OR "vomit*" OR "dis-comfort*" OR "stomach pain*" OR "gastric pain*" OR "ab-dominal pain*" OR "cramp*" OR "lack of energy" OR "head-ache*" OR "safe*" OR "safety*") NOT TS=("animal*" or "pig*" OR "rat*" OR "dog*" OR "bovine*" OR "parenteral*" OR "in-travenous*" OR "in vitro") |

**Supplementary Table 1.** Complete search strategy for PubMed and Web of Science databases.

| **Characteristics** | **Mean ± SD or %** |  | **% of studies** | **n (studies)** |
| --- | --- | --- | --- | --- |
| **Participants per study** | 33.33 ± 35.49 |  |  |  |
| **Age** | 33.58 ± 21.91 |  |  |  |
| **m/f (%)** | 55.14% / 44.86% ^1^ |  |  |  |
| **Preexisting health conditions** |  | **Healthy** (40, 27, 43, 26, 63, 60, 59, 42, 45, 28, 7, 68, 67, 56) | 42.42 | 14 |
|  |  | **Epilepsy** (41, 62, 61, 47, 48, 49, 50, 51, 52, 53, 54) | 33.33 | 11 |
|  |  | **Alzheimer’s Disease** (44, 66, 55) | 9.09 | 3 |
|  |  | **Essential tremor** (64, 46) | 6.06 | 2 |
|  |  | **Obesity** (65) | 3.03 | 1 |
|  |  | **Post-concussion syndrome** (58) | 3.03 | 1 |
|  |  | **Parkinson’s Disease** (57) | 3.03 | 1 |
| ^1^ One study (3.03%) did not report participant sex (63), precluding an exact determination of overall sex distribution. | | | | |

**Supplementary Table 2.** Population characteristics of included studies. Values are reported as mean ± standard deviation or in %.
